# Special role of Foxp3 for the specifically altered microRNAs in Regulatory T cells of HCC patients

**DOI:** 10.1186/1471-2407-14-489

**Published:** 2014-07-07

**Authors:** Long Chen, Huiying Ma, Heng Hu, Lingling Gao, Xuan Wang, Jiaqi Ma, Qiang Gao, Binbin Liu, Guomin Zhou, Chunmin Liang

**Affiliations:** 1 Lab of Tumor Immunology, Department of Anatomy and Histology & Embryology, Shanghai Medical College of Fudan University, 138 Yixueyuan Road, 200032 Shanghai, PR China; 2 Liver Cancer Institute, Zhongshan Hospital, Shanghai Medical College; Key laboratory of Carcinogenesis and Cancer Invasion, Ministry of Education, Fudan University, 136 Yixueyuan Road, 200032 Shanghai, PR China

**Keywords:** Regulatory T cells, Hepatocellular carcinoma, microRNAs array, Foxp3 RNA interference, Bioinformatics

## Abstract

**Background:**

Regulatory T cells (Tregs) exhibit functional abnormalities in the context of hepatocellular carcinoma (HCC). The microRNAs (miRNAs) are identified as the key modulators in Tregs. This study was to explore whether the expression profiles of miRNAs of Tregs were different in HCC-activated Tregs and whether Foxp3 had special effects on them.

**Methods:**

We isolated HCC-activated Tregs from mice bearing HCC and compared the expression profiles of miRNAs between HCC-activated Tregs and control Tregs by microarray. RNA interference against Foxp3 was also performed through transfection of synthetic siRNAs to Tregs for analyzing the effect of Foxp3 on the expression of miRNAs. Tregs isolated from HCC patients (n = 12) and healthy controls (n = 7) were used for validation of the differentially expressed miRNAs. Finally, bioinformatic analysis was applied to infer their possible roles.

**Results:**

We found nine specifically altered miRNAs in HCC-activated Tregs from the murine model. After transfection with siRNAs against Foxp3, control Tregs showed obvious reduction of Foxp3 and five miRNAs were significantly changed; HCC-activated Tregs exhibited a slight reduction of Foxp3 with three miRNAs significantly changed. Tregs from HCC patients and healthy controls finally confirmed the up-regulation of four miRNAs (hsa-miR-182-5p, hsa-miR-214-3p, hsa-miR-129-5p and hsa-miR-30b-5p). Following bioinformatic analysis suggested these altered miRNAs would target eight important signaling pathways that could affect the functions of Tregs.

**Conclusions:**

Our studies provided the first evidence that Tregs in HCC had the specifically altered expression of miRNAs, which was affected by Foxp3. These results are useful both in finding new biomarkers and in further exploring the functions of Tregs in HCC patients.

## Background

Hepatocellular carcinoma (HCC) is the fifth most common cancer with relatively poor overall survival worldwide [1]. Accumulating evidence implies that CD4^+^ CD25^+^ Foxp3^+^ regulatory T cells (Tregs) are the critical factor affecting the progression and prognosis of HCC [2,3]. HCC patients have increased Tregs in peripheral blood, ascites and tumor tissue [4,5] and this prevalence of Tregs correlates with tumor stage and patients survival [6-8]. Not only the number but also the functional phenotypes of Tregs are found abnormal in HCC. Tregs in peripheral blood of HCC patients preferentially up-regulate CCR6, which facilitates their migration to tumor sites [9]. HCC-activated Tregs also express high levels of glucocorticoid-induced tumor necrosis factor receptor (GITR) and the inducible T cell co-stimulator (ICOS), both of which are key mediators for the suppressive function of Tregs [10]. In addition, the enhanced suppressive function of Tregs is confirmed by different studies in HCC patients [10-12].

Tregs constitute the key components in tumor immune suppression [13]. Recent studies demonstrate that Tregs are widely modulated by the single-stranded microRNAs (miRNAs) [14,15]. Depleting Dicer, a key enzyme in the maturation of miRNAs, causes diminished Tregs and compromises their suppressive function [16]. Following studies demonstrate that miR-21 enhances the expression of Foxp3 while miR-31 suppresses it in human Tregs [17]. miR-155, modulated by Foxp3, guarantees competitive fitness of Tregs to the IL-2 signal by targeting suppressor of cytokine signaling 1 (SOCS1) protein [18,19]. Deficiency of miR-146a, another recently confirmed microRNA in Tregs, leads to increased Tregs with an impaired function [20].

However, it is still unclear at present for the following questions in liver cancer research. Firstly, it is still unknown that whether the expression profiles of miRNAs of Tregs are different between control Tregs and HCC-activated Tregs. Secondly, it is worthy to demonstrate whether Foxp3 has a special role and influences the expression profiles of miRNAs of Tregs in HCC. Thirdly, if there are any specifically altered miRNAs in Tregs in HCC, do they affect the functions of Tregs in HCC? It is also need to be further explored. In this study, we used Tregs from both the murine HCC model and HCC patients to answer these questions. We found nine miRNAs differentially expressed in HCC-activated Tregs and alterations of these miRNAs were specific to HCC-activated Tregs in the murine model. Foxp3 affected the expression of these miRNAs. Tregs isolated from human blood confirmed that four miRNAs up-regulated in HCC patients. To our knowledge, this study was the first attempt to characterize miRNAs perturbations and the consequences in Tregs activated by HCC. Our data provided a systemic view on alterations in HCC-activated Tregs, which was not only useful in finding new biomarkers but also in further exploring the functions of Tregs in HCC patients.

## Methods

### Human Tregs separation

Peripheral blood mononuclear cells (PBMCs) were isolated by density gradient sedimentation using Lymphosep (Biowest, France) lymphocyte separation media. CD4^+^ CD25^+^ CD127^-^ Tregs were enriched by human regulatory T cell isolation kit (Miltenyi, German). In brief, non-CD4^+^ and CD127^high^ cells were first depleted with microbeads and then the pre-enriched CD4^+^ CD127^dim^ cells went through positive selection for CD25^+^ T cells. The purity of Tregs was monitored via fluorescence-activated cell sorting (FACS). The participants included HCC patients (n = 12) and matched healthy controls (n = 7). Ethical approval for the use of human subjects was obtained from the Research Ethics Committee of Zhongshan Hospital (Shanghai, PR China), and informed consent was obtained from each participant.

### Cell lines and animals

Hepa 1–6 (CRL-1830), the murine HCC cell line, was obtained from American Type Culture Collection (ATCC, USA) and maintained in DMEM (Biowest) supplemented with 10% FBS (Biowest). C57BL/6 J mice (6 to 8 weeks of age) were purchased from the Chinese Academy of Science and housed at the Animal Maintenance Facility of the Shanghai Medical College, Fudan University. All animal experiments were performed in conformity with the National Institutes of Health Guide for the Care and Use of Laboratory Animals. The Institutional Care of Experimental Animals Committee of Fudan University approved all animal protocols (Permit Number: SYXK 2009–0082).

Hepa 1–6 tumor bearing mice were established as following: mice were injected subcutaneously at the left flank with 3 × 10^6^ Hepa 1–6 cells in 200 μL RPMI 1640 (Biowest) or 200 μL RPMI 1640 alone as control. Three groups of tumor bearing mice and control mice were established (12 mice in each group). Two weeks after inoculation, the mice with visible tumor were sacrificed and spleens were collected for isolation of Tregs using mouse regulatory T cell isolation kit (Miltenyi). In brief, CD4^+^ T cells were enriched by negative selection and then went through positive selection for CD25^+^ T cells. The purity of Tregs was monitored via FACS.

### FACS analysis

For FACS, previously collected Tregs were stained with regulatory T cell Kit (eBioscience, USA). Briefly, cells were first incubated with anti-CD4-FITC and anti-CD25-PE Abs at 4°C for 30 minutes. After washing, cells were treated with fixation/permeabilization buffer and then incubated with anti-Foxp3-PE-Cy5 Abs at 4°C for 30 minutes. Stained cells were subsequently analyzed using an EPICS ALTRA Flow Cytometer (Beckman Coulter, USA).

### Transfection of siRNAs

Validated siRNAs against mouse Foxp3 and AllStars negative control siRNAs were obtained from Qiagen company in a FlexiTube format (Qiagen, German). Four species of siRNAs against Foxp3 (SI01005319, SI01005326, SI01005333 and SI01005340) were mixed in equal amount to generate the Foxp3 siRNAs pool. siRNAs were transfected into HCC-activated Tregs and control Tregs using HiPerFect transfection reagent (Qiagen) according to the manufacturer’s protocol. Tregs were maintained in RPMI 1640 supplemented with 10% heat-inactivated FBS, 5 μg/mL plate-bounded anti-CD3, 5 μg/mL soluble anti-CD28 antibodies (Biolegend, USA) and 40 ng/mL rm IL-2 (PeproTech, USA) at 37°C in humidified atmosphere containing 5% CO_2_ in air. Mixture of 100 nM of siRNAs and 6 μL of HiPerFect reagent were used for each transfection. Then Tregs were harvested for further analysis forty-eight hours after transfection. Two-step qRT-PCR and FACS validated the efficiency of Foxp3 RNA interference (RNAi).

### miRNAs microarray

miRNAs microarray was assisted by Kangcheng Bioscience Service Company (Shanghai). In brief, total RNA was extracted from Tregs of each group using TRIzol (Invitrogen, USA) and miRNeasy mini kit (Qiagen). RNA quality and quantity was measured by NanoDrop-1000 spectrophotometer (Nanodrop Technologies, USA). miRNAs were labeled by the miRCURY™ Hy3™/Hy5™ Power labeling kit (Exiqon, Denmark) and hybridized on the miRCURYTMLNA Array (v.16.0) (Exiqon). The GenePix 4000B microarray scanner (Molecular Devices, USA) scanned the slides. After normalization, volcano plot was used to identify the differentially expressed miRNAs (fold change ≥ 1.5 and *P*-value ≤ 0.05 fold change: the ratio of normalized intensities, HCC-activated Tregs *vs.* control Tregs; *P*-value: *t*-test results between groups). Three independent arrays were performed for HCC-activated Tregs and control Tregs. One array was performed for HCC-activated Tregs transfected with siRNAs against Foxp3 or control siRNAs respectively; one array was performed for control Tregs transfected with siRNAs against Foxp3 or control siRNAs respectively.

### Real-time PCR

Total RNA was extracted from Tregs using TRIzol (Invitrogen) and miRNeasy mini kit (Qiagen). RNA quality and quantity was measured by NanoDrop-1000 spectrophotometer. miRNAs were transcribed to cDNA by cDNA Synthesis Kit (Epicentre, USA) with specific reverse transcription primers (The primers were listed in Additional file 1: Table S1 ). Then the cDNA was used for real time-PCR by miScript SYBRGreen PCR Kit (Qiagen) with specific primers (The primers were listed in Additional file 2: Table S2). For quantification of Foxp3, the cDNA was first synthesized by cDNA Synthesis Kit with Oligo(dT)_21_ primer, and the real time-PCR was performed by miScript SYBRGreen PCR Kit with specific primers (Invitrogen, the primers were listed in Additional file 2: Table S2. The expression levels of miRNAs were presented as 2^-Δ Δ Ct^ or 2^-Δ Ct^ relative to U6 levels and the levels of mRNA were presented as 2^-Δ Δ Ct^ relative to GAPDH levels.

### Bioinformatic analysis

We predicted target genes using the online algorithm TargetScan (Release 6.2, http://www.targetscan.org) [21]. The network of miRNAs and target genes was constructed in the software Exploratory Gene Association Networks (EGAN) [22]. In brief, the total target genes lists were imported into EGAN and further filtered based on Tregs MeSH term (T-Lymphocytes, regulatory). Target genes with direct relation with Tregs MeSH term were presented in the network and further analyzed for functional enrichment. Pathway enrichment was based on the pathways database Kyoto Encyclopedia of Genes and Genomes (KEGG, http://www.genome.jp/kegg/) through DAVID Bioinformatics Resources 6.7 ( http://david.abcc.ncifcrf.gov/home.jsp) [23].

### Statistical analysis

Microarray data of miRNAs was analyzed by volcano plot and the filtering criteria were set at expression fold change ≥ 1.5 and *P*-value ≤ 0.05. Unsupervised hierarchical clustering was performed by MultiExperiment Viewer software (v4.8.1, USA). qRT-PCR validation data was analyzed by Mann–Whitney test. Statistical significance was set at *P*-value < 0.05 (*) or *P-* value < 0.01 (**).

## Results

### miRNAs were differentially and specifically expressed in HCC-activated Tregs

**Figure 1:**
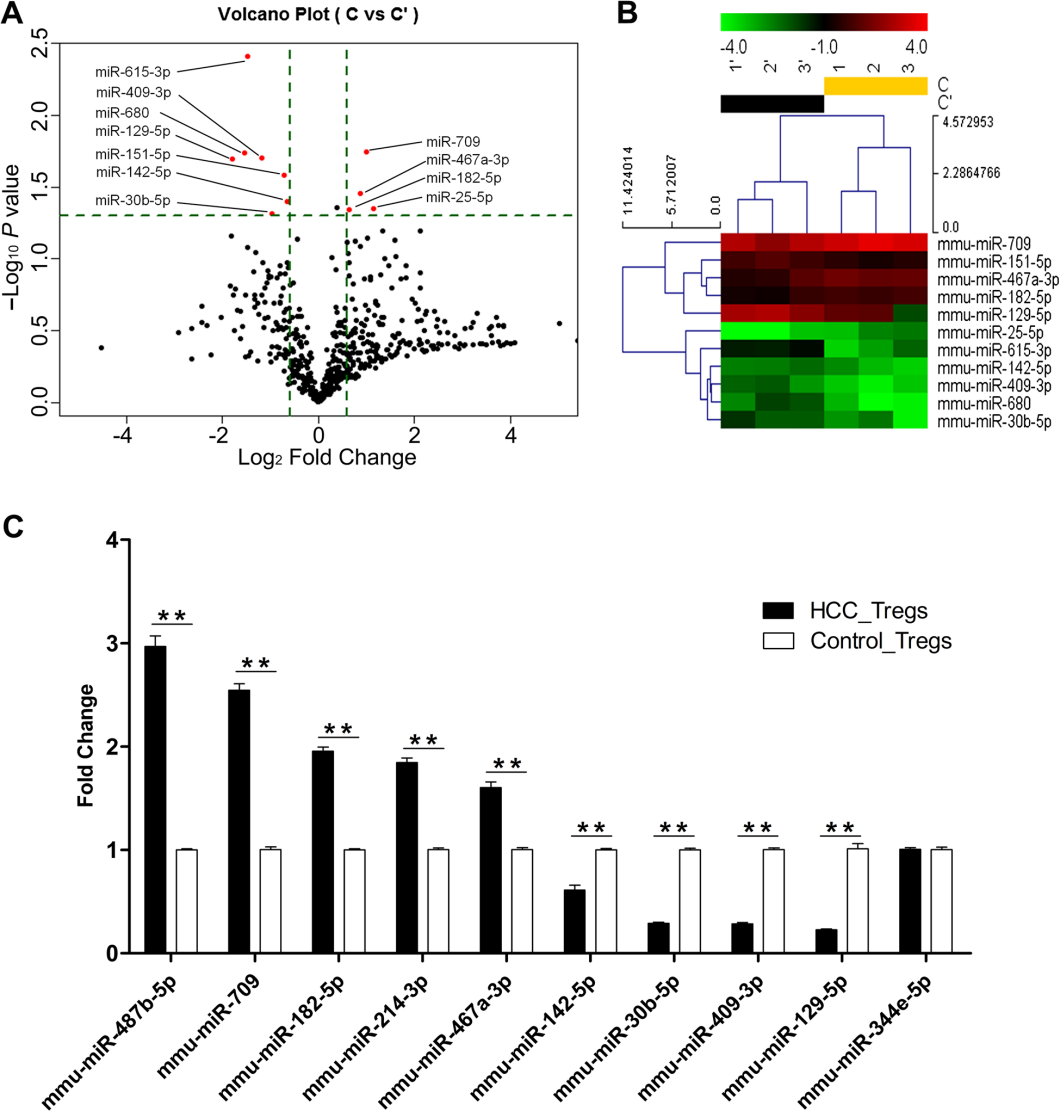
**miRNAs differentially expressed in HCC-activated Tregs. (A)** Differentially expressed miRNAs identified in the microarray. Eleven miRNAs (red plots) passed the volcano plot filtering. **(B)** Unsupervised hierarchical clustering of differentially expressed miRNAs. Scale bar: up-regulated (red), down-regulated (green). Log2 transformed data were used. C: HCC-activated Tregs (three replicates: 1, 2, 3); C’: control Tregs (three replicates: 1’, 2’, 3’). **(C)** Validation of the differentially expressed miRNAs by qRT-PCR. Values were presented as mean ± SEM from three independent experiments performed in triplicates and were analyzed using the two-tailed Mann–Whitney test. **P < 0.01 for indicated comparison.

Tregs were isolated from spleens of mice and the purity of Tregs was examined by fluorescence-activated cell sorting (FACS); only the cells with the purity above 95% (data not shown) were further processed for microarray analysis. Differentially expressed miRNAs in HCC-activated Tregs were selected by volcano plot filtering (fold change ≥ 1.5 and *P*-value ≤ 0.05). Eleven miRNAs were identified as shown in Figure 1A. There were four up-regulated miRNAs (mmu-miR-709, mmu-miR-467a-3p, mmu-miR-182-5p and mmu-miR-25-5p) and seven down-regulated miRNAs (mmu-miR-615-3p, mmu-miR-409-3p, mmu-miR-680, mmu-miR-129-5p, mmu-miR-151-5p, mmu-miR-142-5p and mmu-miR-30b-5p), as the values presented in Table 1. Then we performed unsupervised hierarchical clustering of the eleven miRNAs. We found these miRNAs clearly discriminated the HCC-activated Tregs from control Tregs, as shown in Figure 1B.

**Table 1:** Differentially expressed miRNAs in HCC-activated Tregs

**Name**	**Fold change (Tumor *vs. *** **Control)**	***P*-value**
mmu-miR-25-5p	2.21	0.04
mmu-miR-709	1.98	0.02
mmu-miR-467a-3p	1.82	0.04
mmu-miR-182-5p	1.54	0.05
mmu-miR-129-5p	0.29	0.02
mmu-miR-680	0.34	0.02
mmu-miR-615-3p	0.36	0.00
mmu-miR-409-3p	0.44	0.02
mmu-miR-30b-5p	0.51	0.05
mmu-miR-151-5p	0.61	0.03
mmu-miR-142-5p	0.63	0.04

By TargetScan, we found that mmu-miR-25-5p, mmu-miR-615-3p, mmu-miR-151-5p and mmu-miR-680 had few target genes directly relating with Tregs in MeSH database, so we excluded the four miRNAs for further exploration. As mmu-miR-155 and mmu-let-7i have been well documented in T cells [19,24,25], we observed that mmu-miR-487b-5p and mmu-miR-214-3p were classified into the same group with mmu-miR-155 and mmu-let-7i respectively after hierarchical clustering (data not shown). Therefore, we also included these two miRNAs for further validation. To verify the credibility of qRT-PCR validation, we included the miR-344e-5p as a negative control as it did not pass volcano plot filtering (fold change = 1.85, *P*-value = 0.54) in microarray. Among the ten miRNAs validated by qRT-PCR, we found that *mmu-miR-487b-5p, mmu-miR-709, mmu-miR-182-5p, mmu-miR-214-3p* and *mmu-miR-467a-3p* were up-regulated in HCC-activated Tregs, *mmu-miR-142-5p, mmu-miR-30b-5p, mmu-miR-409-3p* and *mmu-miR-129-5p* were down-regulated (*P* < 0.01), while miR-344e-5p did not change significantly, as shown in Figure 1C.

### Foxp3 was involved in regulating the miRNAs

**Figure 2:**
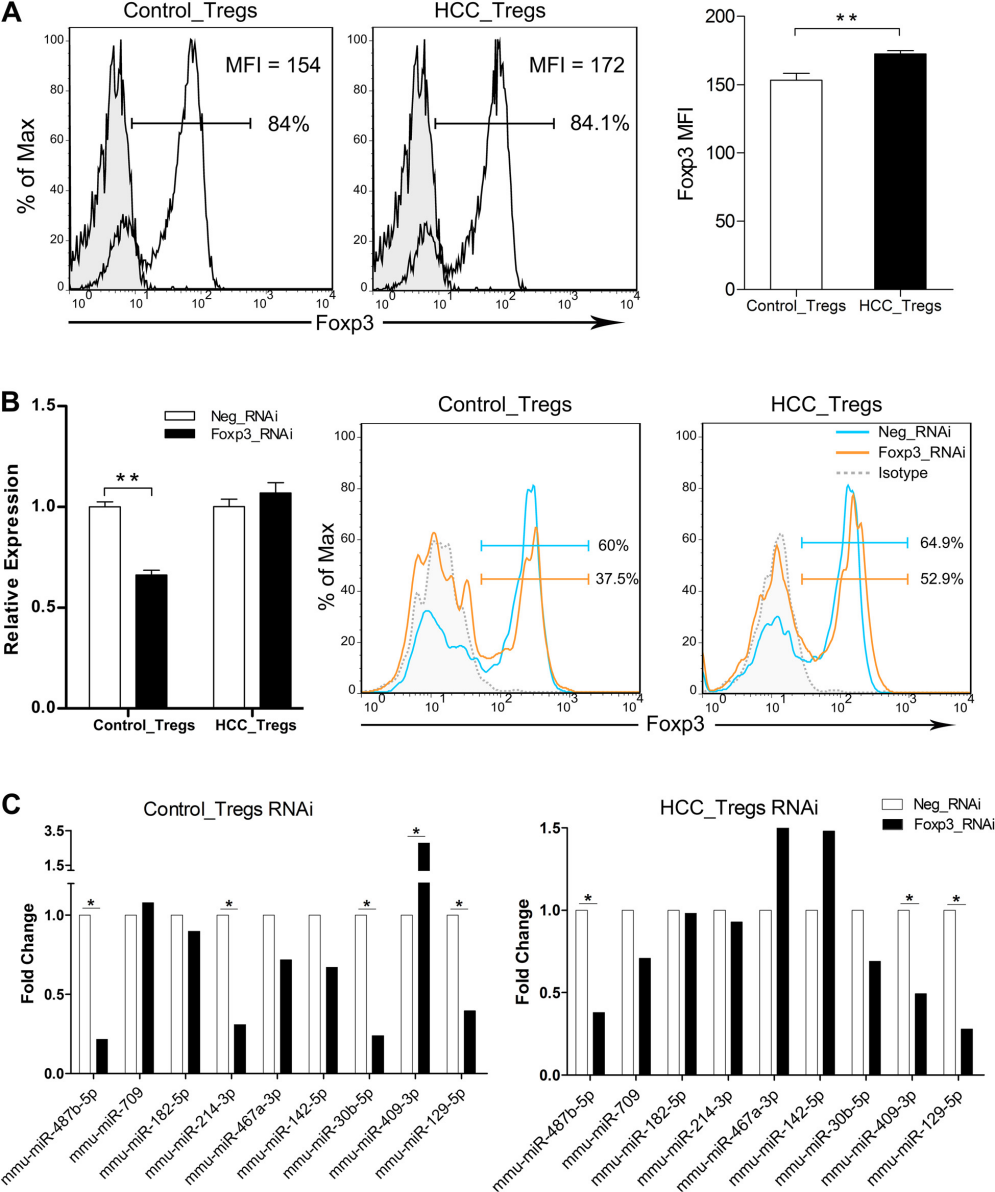
**Special modulation of the miRNAs by Foxp3. (A)** Representative FACS plots of Foxp3 were shown in control Tregs and HCC-activated Tregs (left). Mean fluorescence intensity (MFI) was included in each of the panels. Summary of the Foxp3 MFI was presented (right) in control Tregs and HCC-activated Tregs. **(B)** Tregs were transfected with Foxp3-specific siRNAs or negative controls and forty-eight hours later cells were harvested. Expression levels of Foxp3 were determined by qRT-PCR (left) and representative FACS plots were shown in control Tregs and HCC-activated Tregs (right). Results in **(A)** and **(B)** were presented as mean ± SEM of three independent experiments performed in triplicates and analyzed by the two-tailed Student’s *t*-test. **P < 0.01 for indicated comparison. **(C)** Expression patterns of the nine miRNAs after Foxp3 silencing in control Tregs (left) and HCC-activated Tregs (right). The expression levels of each miRNAs after Foxp3 silencing were shown as fold change relative to the negative control siRNAs. Data were from one microarray. * fold change > 1.5 for indicated comparison. Control_Tregs: Tregs from control mice; HCC_Tregs: Tregs from mice bearing Hepa 1–6; Neg_RNAi: Tregs transfected with negative control siRNAs; Foxp3_RNAi: Tregs transfected with siRNAs against Foxp3.

As Foxp3 is the master regulator in Tregs, it prompts us to check whether these miRNAs would be specifically affected by Foxp3. We compared the mean fluorescence intensity (MFI) of Foxp3 in Tregs after magnetic sorting. FACS results showed that the sorted Tregs had comparable percentages of Foxp3 positive cells; however, the MFI was higher in HCC-activated Tregs compared with control Tregs (Figure 2A). We transfected Tregs with siRNAs against Foxp3 or negative controls and forty-eight hours we determined the efficiency of silencing. The qRT-PCR data demonstrated that mRNA levels of Foxp3 reduced significantly (34%) in control Tregs, whereas the levels did not change significantly in HCC-activated Tregs (Figure 2B, left). While the FACS results showed the protein levels of Foxp3 reduced in both groups, we observed a more potent decrease of Foxp3 in control Tregs after siRNA silencing (Figure 2B, right). Then we examined the expression levels of the nine miRNAs in Tregs from our microarray data after transfection. In control Tregs, *mmu-miR-487b-5p, mmu-miR-214-3p, mmu-miR-30b-5p* and *mmu-miR-129-5p* showed significant down-regulation while *mmu-miR-409-3p* showed significant up-regulation (Figure 2C, left). Compared with control Tregs, although *mmu-miR-487b-5p* and *mmu-miR-129-5p* showed similar down-regulation in HCC-activated Tregs, *mmu-miR-409-3p* was actually significantly down-regulated; *mmu-miR-214-3p* and *mmu-miR-30b-5p* did not exhibit significant changes (Figure 2C, right).

### Expression patterns of the miRNAs in human Tregs

**Figure 3:**
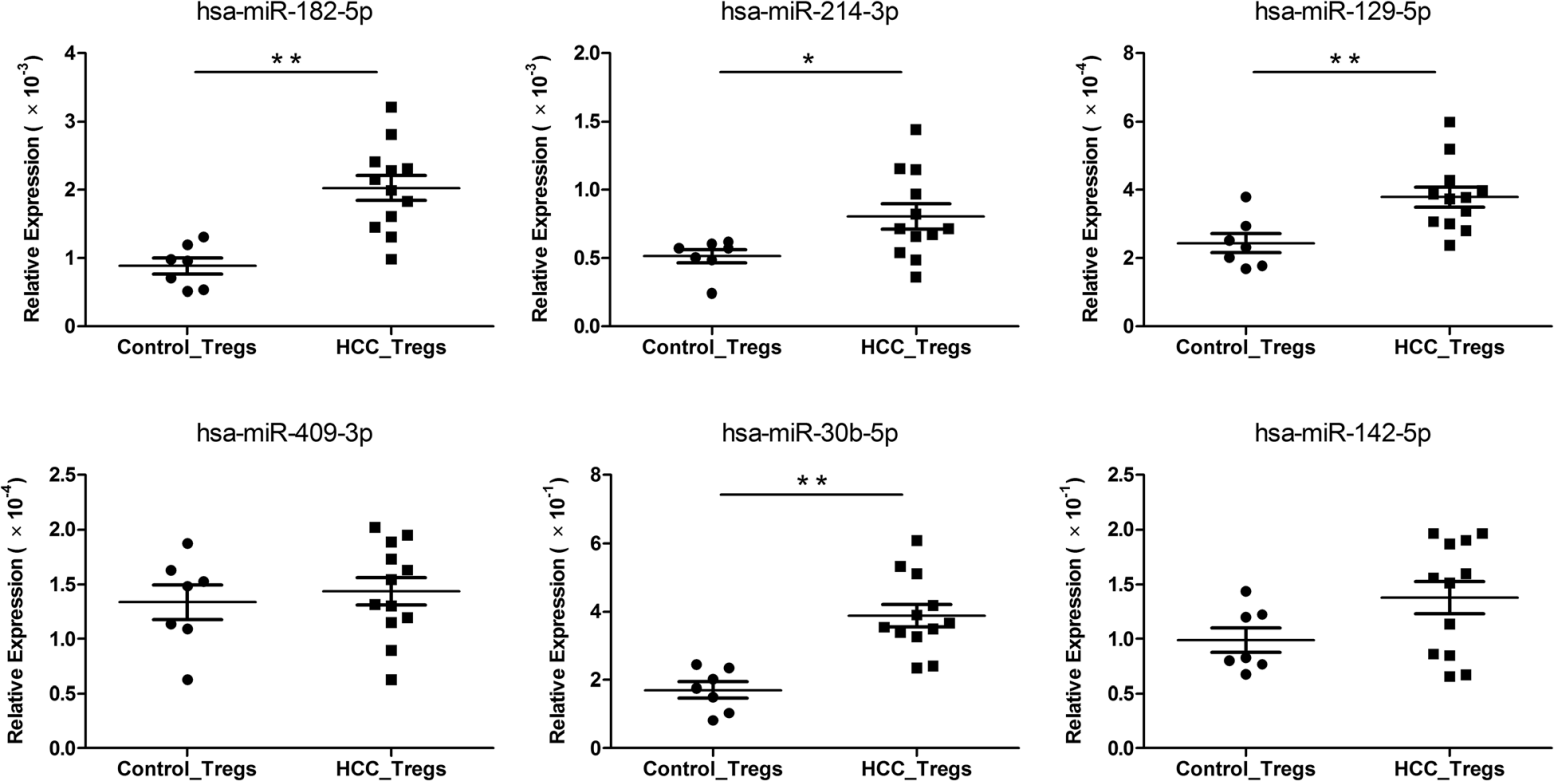
**Expression levels of the miRNAs in Tregs from healthy controls and HCC patients.** Expression levels of six selected miRNAs were determined by qRT-PCR in Tregs sorted from peripheral blood of healthy controls (n = 7) and HCC patients (n = 12). Expression data were normalized to U6 levels. Horizontal lines represented the mean and error bars represented the SEM. Control_Tregs: Tregs from healthy controls; HCC_Tregs: Tregs from HCC patients. Results were analyzed by the two-tailed Mann–Whitney test. *P < 0.05, **P < 0.01 for indicated comparison.

We wondered whether these miRNAs were also differentially expressed in HCC patients. Because *miR-487b-5p, miR-709 and miR-467a-3p* did not express in human tissue (miRBase 19), we checked the expression levels of the rest six miRNAs in Tregs from peripheral blood samples. Compared with the healthy controls, the expression levels of *hsa-miR-182-5p, hsa-miR-214-3p, hsa-miR-129-5p* and *hsa-miR-30b-5p* were significantly up-regulated in Tregs from HCC patients while the *hsa-miR-409-3p* and *hsa-miR-142-5p* did not show significant changes (Figure 3).

### Possible roles of target genes inferred by bioinformatic analysis

**Figure 4:**
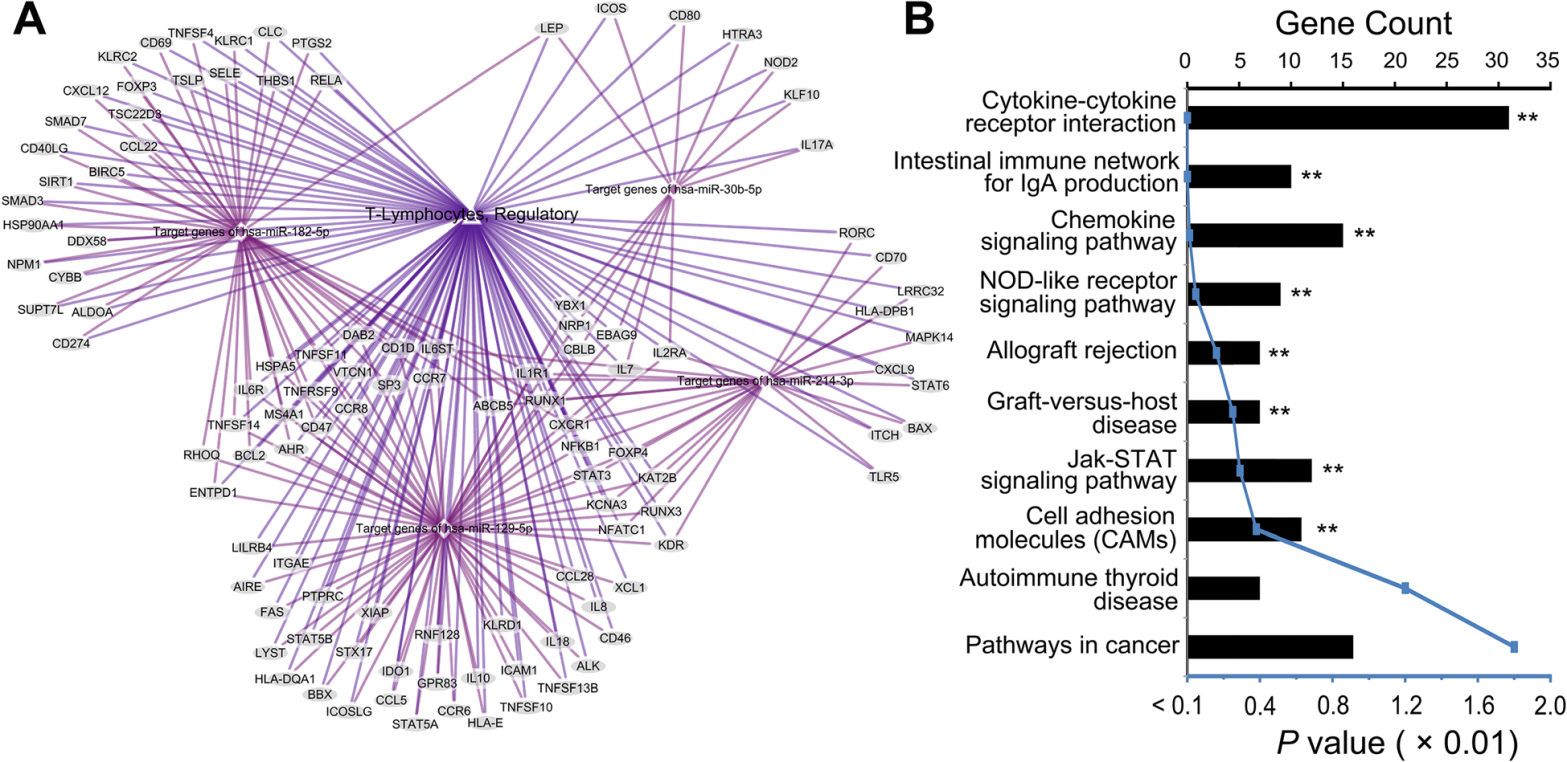
**Bioinformatic analysis of target genes of the four miRNAs. (A)** The network of target genes of the four miRNAs involved in Tregs functions. Each ellipse represented a target gene predicted by TargetScan. The four rhombuses were miRNAs and the triangle was the MeSH term of Tregs. Purple lines indicated genes involved in Tregs according to MeSH database; magenta lines outlined the miRNAs-target genes relations. **(B)** Pathway enrichment of target genes. The y-axis represented pathways enriched based on predicted target genes of the four miRNAs. The histogram indicated gene number in each pathway (upper black x-axis); the curve line indicated the *P* value (lower blue x-axis). **P < 0.01 with the Bonferroni correction.

The functions of these four miRNAs (*hsa-miR-182-5p, hsa-miR-214-3p, hsa-miR-129-5p* and *hsa-miR-30b-5p*) in human Tregs are not clear. Therefore, we applied bioinformatic methods to explore their roles. Target genes of these four miRNAs were predicted by TargetScan 6.2 and all the genes were imported to software EGAN. The total 109 target genes involved in Tregs functions were enriched based on MeSH database and constructed into the network (Figure 4A). These target genes were further analyzed by functional enrichment based on KEGG pathways via DAVID and eight pathways were enriched with statistical significance (Figure 4B). Among these pathways, two pathways were involved in cytokine signaling (*Cytokine-cytokine receptor interaction* and *Jak-STAT signaling pathway*); two pathways were associated with chemotaxis (*Chemokine signaling pathway* and *Cell adhesion molecules (CAMs)*); two pathways were related with immune response to graft (*Allograft rejection* and *Graft-versus-host disease*). The other two pathways were *Intestinal immune network for IgA production* and *NOD-like receptor signaling pathway*.

## Discussion

In this study, we found nine differentially expressed miRNAs in Tregs from the murine HCC model, which were modulated by Foxp3, and validated four of them in Tregs from HCC patients. Bioinformatic analysis suggested the four miRNAs had important roles by targeting genes in several pathways affecting Tregs functions.

It has been reported that alterations of miRNAs in CD4^+^ T cells and Tregs are correlated with certain activation states and diseases [26-28]. Our array data found a group of differentially expressed miRNAs in Tregs from the murine HCC model. Interestingly, the expression patterns of these miRNAs were specific to HCC-activated Tregs. It is supposed that Tregs suppress the immune response in a context dependent way [29-31]. For example, depleting the signal transducer and activator of transcription 3 (*STAT3)*, which is essential for proper development of Th17 cells, results in failure of Tregs to suppress Th17 cell-mediated disease [32]. Assisted by bioinformatic analysis, we selected ten miRNAs for qRT-PCR validation. The validation data were consistent with array results, indicating the up-regulation of five miRNAs and down-regulation of four miRNAs. Because miRNAs can simultaneously regulate a large number of genes, they might be the proper candidate for this context dependent modulation. We proposed that the specific tumor antigen, tumor associated antigen or tumor derived signals might contribute to the unique alterations in HCC-activated Tregs, which was possibly mediated by miRNAs.

Because Foxp3 is the crucial transcription factor in Tregs, we wondered whether these altered miRNAs were affected by Foxp3. As Foxp3 has been reported to be up-regulated in activated Tregs in some reports [33,34], we first compared the MFI of Foxp3 in control Tregs and HCC-activated Tregs. Consisted with previous reports, we found higher MFI in HCC-activated Tregs. After transfection of siRNAs against Foxp3, we determined the mRNA and protein levels of Foxp3. Control Tregs showed significant down-regulation of Foxp3 after silencing at both the mRNA and protein levels; however, HCC-activated Tregs showed slight down-regulation of Foxp3 protein, and no significant changes of Foxp3 mRNA. So the increased expression of Foxp3 in HCC-activated Tregs might involve in the reduced efficacy of Foxp3 RNAi. One report recently demonstrates that under certain inflammatory milieu Foxp3 undergoes phosphorylation, which affects its stability and function [35]. This report and our present data support the hypothesis that both the modification state and the expression level of Foxp3 in HCC-activated Tregs can affect the efficiency of Foxp3 silencing.

We also found five miRNAs showed significant fold changes after Foxp3 RNAi in control Tregs, among which three miRNAs showed significant fold change in HCC-activated Tregs. Two miRNAs (*mmu-miR-214-3p* and *mmu-miR-30b-5p*) were significantly changed only in control Tregs. Our findings were consistent with previous studies, which have demonstrated that Foxp3 modulates the expression of miR-155 that maintains the functions of Tregs [18]. Considering the relatively low silencing efficacy of Foxp3 in HCC-activated Tregs, we thought the modulation of these two miRNAs were not so sensitive to Foxp3 levels compared with that of the other three miRNAs.

We further validated the expression levels of these miRNAs in Tregs from HCC patients and healthy controls. Because *miR-487b-5p, miR-709 and miR-467a-3p* did not express in human tissue (miRBase 19), we validated the expression levels of the rest six miRNAs. Four miRNAs showed significant changes in HCC-activated Tregs compared with healthy controls. Interestingly, compared with data from the murine model, two of the four miRNAs (*hsa-miR-182-5p* and *hsa-miR-214-3p*) showed the similar up-regulation while the other two miRNAs (*hsa-miR-129-5p* and *hsa-miR-30b-5p*) showed reverse changes. We were not sure whether this discrepancy was due to the differences of species or HCC tumor models. Further experiments were need to clarify this question.

The functions of four up-regulated miRNAs were not reported in Tregs and we performed bioinformatic analysis to infer their possible roles. The target genes with direct relations with Tregs MeSH term were found significantly involved in eight pathways. Two of them were cytokine signaling, including genes *IL6ST*, *IL6R*, *STAT3* and *IL17A* which have been reported to facilitate the differentiation of Th17 by inhibiting Tregs induction [36-38]. Up-regulation of these miRNAs might break the balance between Th17 and Tregs and finally accelerate the production of Tregs, which contributes to the abnormal homeostasis of Tregs in HCC [9,39,40]. Another two pathways related with chemotaxis, which has been reported to be critical for the migration and distribution of Tregs in HCC. CC chemokine receptor 6 (CCR6) axis has an important role in recruiting Tregs to tumor sites in HCC [9]; TGF-beta and macrophage-derived chemokine (CCL22) signaling pathways induce aggregation of Tregs at the tumor sites in HCC too [41]. These two pathways included genes in chemotaxis and cell adhesion such as *CCR6, CCR7, CXCR1, SELE* and *ICOS*[9,42-44]. These alterations might contribute to the abnormal distribution of Tregs in HCC at the tumor sites. We also found two pathways relating with immune response to allograft (*Allograft rejection* and *Graft-versus-host disease*). Although it is well established that Tregs are critical for maintaining the tolerance to allograft [45-47], it is not clear whether the same genes or pathways work similarly in Tregs during the progression of HCC. These new clues needed further exploring. IgA production is essential for the intestinal homeostasis, in which Tregs are indispensable via secretion of TGF-beta [48,49]. It was possible that Tregs applied the same mechanism via TGF-beta in HCC. NOD-like receptor is one of the conserved pattern-recognition receptors (PRRs) which included Toll-like receptors (TLRs) [50]. Previous studies have demonstrated that TLR1, TLR2, TLR4 and TLR7 have important functions in Tregs [51-54] and we proposed that NOD-like receptors were new key PRRs in the context of HCC.

## Conclusions

In summary, we confirmed nine differentially expressed miRNAs in Tregs from the HCC murine model. These miRNAs exhibited a specific expression pattern in HCC-activated Tregs and were affected by Foxp3. Four miRNAs were finally found to be up-regulated in HCC patients for the first time. Bioinformatic analysis indicated the four miRNAs (*hsa-miR-182-5p, hsa-miR-214-3p*, *hsa-miR-129-5p and hsa-miR-30b-5p*) targeted eight signaling pathways involved in Tregs. These results provided interesting information on the intrinsic functional changes occurred in HCC-activated Tregs, which were worthy of further exploration.

## Abbreviations

HCC: Hepatocellular carcinoma; Tregs: Regulatory T cells; miRNAs: microRNAs; RNAi: RNA interference; MFI: Mean fluorescence intensity; FACS: Fluorescence-activated cell sorting.

## Competing interests

The authors declare that they have no competing interests.

## Authors’ contributions

LC, HM and HH performed the experiments, interpreted the findings and prepared the manuscript. LG, XW and JM assisted in animals’ maintenance. QG prepared blood samples and obtained informed consent from the patients. BL performed the statistical analysis. GZ participated in the design, CL conceived of the study, participated in the design, and assisted with data interpretation and manuscript writing. All authors read and approved the final manuscript.

## Pre-publication history

The pre-publication history for this paper can be accessed here:

http://www.biomedcentral.com/1471-2407/14/489/prepub

## Supplementary Material

Additonal file 1: Table S1 Primers for Reverse Transcription. Click here for file

Additional file 2: Table S2 Primers for Real-time PCR. Click here for file
