# Supplementary material for: Special role of Foxp3 for the specifically altered microRNAs in Regulatory T cells of HCC patients
Source: BMC Cancer. 2014 Jul 7;14:489. doi: 10.1186/1471-2407-14-489 (PMC4099493; doi:10.1186/1471-2407-14-489)
Supplement: Additonal file 1: Table S1 — Primers for Reverse Transcription. [file 1471-2407-14-489-S1.doc]

| **Additional file 2: Table S1. Primers for Reverse Transcription** | |
| --- | --- |
| **Name** | **Primer sequence** |
| U6 | 5’-CGCTTCACGAATTTGCGTGTCAT-3’ |
| mmu-miR-487b-5p | 5’-GTCGTATCCAGTGCGTGTCGTGGAGTCGGCAATTGCACTGGATACGACCGAAGA-3’ |
| mmu-miR-709 | 5’-GTCGTATCCAGTGCGTGTCGTGGAGTCGGCAATTGCACTGGATACGACTCCTCC-3’ |
| mmu-miR-182-5p | 5’-GTCGTATCCAGTGCGTGTCGTGGAGTCGGCAATTGCACTGGATACGACCGGTGT-3’ |
| mmu-miR-214-3p | 5’-GTCGTATCCAGTGCGTGTCGTGGAGTCGGCAATTGCACTGGATACGACACTGCC-3’ |
| mmu-miR-467a-3p | 5’-GTCGTATCCAGTGCGTGTCGTGGAGTCGGCAATTGCACTGGATACGACTGTAGG-3’ |
| mmu-miR-142-5p | 5’-GTCGTATCCAGTGCGTGTCGTGGAGTCGGCAATTGCACTGGATACGACAGTAGT-3’ |
| mmu-miR-30b-5p | 5’-GTCGTATCCAGTGCGTGTCGTGGAGTCGGCAATTGCACTGGATACGACAGCTGA-3’ |
| mmu-miR-409-3p | 5’-GTCGTATCCAGTGCGTGTCGTGGAGTCGGCAATTGCACTGGATACGACAGGGGT-3’ |
| mmu-miR-129-5p | 5’-GTCGTATCCAGTGCGTGTCGTGGAGTCGGCAATTGCACTGGATACGACGCAAGC-3’ |
| mmu-miR-344e-5p | 5’-GTCGTATCCAGTGCGTGTCGTGGAGTCGGCAATTGCACTGGATACGACGGAATA-3’ |
| hsa-miR-182-5p | 5’-GTCGTATCCAGTGCGTGTCGTGGAGTCGGCAATTGCACTGGATACGACAGTGTGA-3’ |
| hsa-miR-214-3p | 5’-GTCGTATCCAGTGCGTGTCGTGGAGTCGGCAATTGCACTGGATACGACACTGCC-3’ |
| hsa-miR-129-5p | 5’-GTCGTATCCAGTGCGTGTCGTGGAGTCGGCAATTGCACTGGATACGACGCAAGC-3’ |
| hsa-miR-409-3p | 5’-GTCGTATCCAGTGCGTGTCGTGGAGTCGGCAATTGCACTGGATACGACAGGGGT-3’ |
| hsa-miR-30b-5p | 5’-GTCGTATCCAGTGCGTGTCGTGGAGTCGGCAATTGCACTGGATACGACAGCTGA-3’ |
| hsa-miR-142-5p | 5’-GTCGTATCCAGTGCGTGTCGTGGAGTCGGCAATTGCACTGGATACGACAGTAGTGC-3’ |
