# Supplementary material for: Special role of Foxp3 for the specifically altered microRNAs in Regulatory T cells of HCC patients
Source: BMC Cancer. 2014 Jul 7;14:489. doi: 10.1186/1471-2407-14-489 (PMC4099493; doi:10.1186/1471-2407-14-489)
Supplement: Additional file 2: Table S2 — Primers for Real-time PCR. [file 1471-2407-14-489-S2.doc]

| **Additional file 2: Table S2. Primers for Real-time PCR** | | |
| --- | --- | --- |
| Name | Primer sequence | Fragment length ( bp ) |
| U6 | F: 5’-GCTTCGGCAGCACATATACTAAAAT-3’ R: 5’-CGCTTCACGAATTTGCGTGTCAT-3’ | 89 |
| mmu-miR-487b-5p | F: 5'-GGGTGGTTATCCCTGTCC-3' R: 5'-CAGTGCGTGTCGTGGAGT-3' | 64 |
| mmu-miR-709 | F: 5'-GGAAGGAGGCAGAGGC-3' R: 5'-CAGTGCGTGTCGTGGAG-3' | 63 |
| mmu-miR-182-5p | F: 5'-TTTGGCAATGGTAGAACTC-3' R: 5'-CAGTGCGTGTCGTGGA-3' | 65 |
| mmu-miR-214-3p | F: 5'-GGACAGCAGGCACAGACA-3' R: 5'-CAGTGCGTGTCGTGGAGT-3' | 64 |
| mmu-miR-467a-3p | F: 5'-GGGGCATATACATACACACA-3' R: 5'-CAGTGCGTGTCGTGGA-3' | 66 |
| mmu-miR-142-5p | F: 5'-GGGGCATAAAGTAGAAAGC-3' R: 5'-CAGTGCGTGTCGTGGAG-3' | 65 |
| mmu-miR-30b-5p | F: 5'-GGGGGCTAAACATCCTACAC-3' R: 5'-CAGTGCGTGTCGTGGAGT-3' | 66 |
| mmu-miR-409-3p | F: 5'-GGGAATGTTGCTCGGTGA-3' R: 5'-CAGTGCGTGTCGTGGAGT-3' | 64 |
| mmu-miR-129-5p | F: 5'-GGCTTTTTGCGGTCTGG-3' R: 5'-CAGTGCGTGTCGTGGAGT-3' | 63 |
| mmu-miR-344e-5p | F: 5'-GGGCAGGCTTCTGGCTA-3' R: 5'-CAGTGCGTGTCGTGGAGT-3' | 63 |
| hsa-miR-182-5p | F: 5’-GCTTTGGCAATGGTAGAAC-3’ R: 5’-CAGTGCGTGTCGTGGAG-3’ | 66 |
| hsa-miR-214-3p | F: 5’-GACAGCAGGCACAGACA-3’ R: 5’-TGCGTGTCGTGGAGTC-3’ | 61 |
| hsa-miR-129-5p | F: 5’-GCTTTTTGCGGTCTGG-3’ R: 5’-TGCGTGTCGTGGAGTC-3’ | 59 |
| hsa-miR-409-3p | F: 5’-GGAATGTTGCTCGGTGA-3’ R: 5’-CAGTGCGTGTCGTGGA-3’ | 63 |
| hsa-miR-30b-5p | F: 5’-GGGGGGTAAACATCCTACAC-3’ R: 5’-CAGTGCGTGTCGTGGAGT-3’ | 66 |
| hsa-miR-142-5p | F: 5’-GGGGCATAAAGTAGAAAGC-3’ R: 5’-CAGTGCGTGTCGTGGAG-3’ | 65 |
| Foxp3 | F: 5’-CCCATCCCCAGGAGTCTTG-3’ R: 5’-CACCATGACTAGGGGCACTGTA-3’ | 184 |
| GAPDH | F: 5’-GTTGTCTCCTGCGACTTCA-3’ R: 5’-GCCCCTCCTGTTATTATGG-3’ | 293 |
